# Mortality Risk in Patients With Cardiac Complications Following Ischemic Stroke: A Report From the Virtual International Stroke Trials Archive

**DOI:** 10.1161/JAHA.124.036799

**Published:** 2024-11-22

**Authors:** Hironori Ishiguchi, Bi Huang, Wahbi K. El‐Bouri, Jesse Dawson, Gregory Y. H. Lip, Azmil H. Abdul‐Rahim, for the VISTA Collaborators, A. Alexandrov, P. M. Bath, E. Bluhmki, N. Bornstein, C. Chen, L. Claesson, J. Curram, S. M. Davis, H‐C. Diener, G. Donnan, M. Fisher, M. Ginsberg, B. Gregson, J. Grotta, W. Hacke, M. G. Hennerici, M. Hommel, M. Kaste, P. Lyden, J. Marler, K. Muir, C. Roffe, R. Sacco, P. Teal, N. Venketasubramanian, N. G. Wahlgren, S. Warach

**Affiliations:** ^1^ Liverpool Centre for Cardiovascular Science at University of Liverpool, Liverpool John Moores University and Liverpool Heart & Chest Hospital Liverpool UK; ^2^ Division of Cardiology, Department of Medicine and Clinical Science Yamaguchi University Graduate School of Medicine Ube Japan; ^3^ Department of Cardiovascular and Metabolic Medicine Institute of Life Course and Medical Sciences, University of Liverpool Liverpool UK; ^4^ School of Cardiovascular and Metabolic Health College of Medical, Veterinary & Life Sciences, University of Glasgow Glasgow UK; ^5^ Danish Centre for Health Services Research, Department of Clinical Medicine Aalborg University Aalborg Denmark; ^6^ Stroke Division, Department Medicine for Older People Mersey and West Lancashire Teaching Hospitals NHS Trust Prescot UK

**Keywords:** ischemic stroke, death, stroke‐heart syndrome, Ischemic Stroke

## Abstract

**Background:**

Cardiac complications may occur in patients following ischemic stroke (stroke‐heart syndrome [SHS]). We investigated the mortality risk in patients with SHS and across the SHS manifestations.

**Methods and Results:**

Data were sought from the VISTA (Virtual International Stroke Trials Archive), an international repository of clinical trials data. We reviewed relevant adverse events and classified patients into 2 cohorts based on the incidence of SHS. The SHS was defined as developing any cardiac complications within 30 days following stroke. Using Cox proportional hazards models, we evaluated the temporal risk dynamics of 90‐day death associated with the day of SHS onset. We also compared the risk of 90‐day death across SHS manifestations, using multivariate analysis. Among 15 054 patients with ischemic stroke (mean age, 69±12 years; 55% men), 1787 (11.8% [95% CI, 11.3–12.3]) developed SHS. The median onset time for SHS was 2 (interquartile range, 1–4) days. The most prevalent manifestation was other arrhythmia/ECG abnormalities, with the incidence rate of 6.5% (95% CI, 6.1–6.9). Patients who developed SHS between 10 and 30 days following stroke had significantly higher risks of death compared with those with SHS within the first 0 to 3 days (adjusted hazard ratio, 1.84 [95% CI, 1.36–2.49]).

In the multivariate‐adjusted analysis, SHS manifested as acute myocardial injury/myocardial injury, heart failure/left ventricular dysfunction, and atrial fibrillation/flutter were associated with the highest risk of death within 90 days after stroke across SHS manifestations excluding cardiorespiratory arrest.

**Conclusions:**

SHS is associated with a high risk of death, with a greater risk observed with delayed SHS onset.

## Graphical abstract

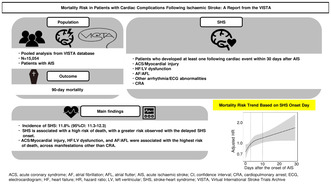

**Nonstandard Abbreviations and Acronyms**
AFL: atrial flutter
AIS: acute ischemic stroke
CRA: cardiorespiratory arrest
SHS: stroke‐heart syndrome
VISTA: Virtual International Stroke Trials Archive

**Clinical Perspective**

**What Is New?**

- Although the incidence of stroke‐heart syndrome peaked within the first 3 days after stroke, the mortality risk increased with later stroke‐heart syndrome onset.
- Among stroke‐heart syndrome manifestations, excluding cardiorespiratory arrest, acute coronary syndrome/myocardial injury, heart failure/left ventricular dysfunction, and atrial fibrillation/flutter were independently associated with 90‐day poststroke death.

**What Are the Clinical Implications?**

- Our study emphasizes the importance of early detection and intervention in stroke‐heart syndrome due to the increasing mortality risk with delayed onset.
- We also highlight the significant impact of acute coronary syndrome/myocardial injury, heart failure/left ventricular dysfunction, and atrial fibrillation/flutter on the mortality rate, underscoring the need for clinicians to closely monitor and manage these complications.

The management of acute ischemic stroke (AIS) remains a major cause of death and morbidity, despite recent advances in therapeutic strategies.^1^, ^2^ Recent research has shown that ≈20% of patients with AIS developed cardiac complications, adversely affecting their prognosis in both the short and long term.^3^, ^4^, ^5^

The concept of “stroke‐heart syndrome” (SHS) encapsulates the cardiac complications following AIS, characterized by the emergence of new heart conditions or the exacerbation of preexisting cardiac diseases within 30 days after AIS.^6^ SHS manifestations include acute myocardial infarction; acute myocardial injury; heart failure; systolic dysfunction, including Takotsubo syndrome; arrhythmias, including ECG abnormalities and atrial fibrillation (AF); and sudden cardiac death.^4^, ^6^ The underlying mechanisms of SHS are thought to be autonomic disturbances, neurohormonal malfunctions, and neuroinflammation, stemming from the disrupted brain–heart interaction following cerebral injury from the acute stroke.^4^, ^6^, ^7^

Although research has highlighted the adverse effects of SHS, the time course of its incidence and its prognostic implications during the acute phase of AIS remain incompletely understood.^8^, ^9^ Evidence suggests that the onset of SHS and its certain manifestations typically peak within a few days following AIS onset.^3^, ^10^, ^11^ Nevertheless, it is still unclear whether this peak in SHS development is directly associated with an increased mortality risk. This uncertainty highlights the need to explore the temporal dynamics of mortality risk relative to the timing of SHS onset. Moreover, while individual studies have documented the detrimental impacts of each SHS manifestation, a detailed comparative analysis among these different manifestations is yet to be undertaken.

We aimed to explore the temporal risk dynamics of death associated with the timing of SHS onset and to compare the impact of various manifestations of SHS on outcomes, using adjudicated clinical events in randomized trials.

## Methods

### Data Resource

We conducted a retrospective analysis on individual patient data, pooled from randomized clinical trials, available within the VISTA (Virtual International Stroke Trials Archive^12^; https://www.virtualtrialsarchives.org/vista/). VISTA is a collaborative registry that collates and provides access to completed acute stroke trials' data (from year 1998 to 2010), anonymized in relation to patient and trial identity, for novel exploratory analyses.^13^ VISTA has institutional ethical approval (University of Glasgow UK, College of Medical Veterinary and Life Sciences' ethics committee) for the use of fully anonymized data for novel research purposes. Informed consent was not sought for the present study because it uses pooled, anonymized data from a clinical trial resource. Anonymized patient‐level data from VISTA are available upon reasonable request through its online platform. It is worth noting that VISTA data do not include trials of thrombolysis therapy, per se, although thrombolysis was commonly used as standard therapy, where appropriate. All patients with stroke were treated as per institutional practice and stroke guidelines acceptable at the point of trial conduct.^13^ The conduct and reporting of our analysis are in accordance with the Strengthening the Reporting of Observational Studies in Epidemiology guidelines for cohort studies.^14^

We selected patients with ischemic stroke who had been randomized to receive placebo or any drug now known to possess no confirmed action on stroke outcome. We included patients for whom we had baseline demographics and outcome information: individual components of the baseline National Institutes of Health Stroke Scale score, age, sex, comorbidities, occurrence of adverse events, serious adverse events, and death by 90 days. The starting point for death was set as the onset of stroke.

### Study Design

We evaluated the incidence of SHS and each of its manifestations within our cohort. We identified patients with SHS, that is, patients who developed at least 1 of the following manifestations within 30 days following stroke: acute coronary syndrome (ACS; including acute myocardial infarction and unstable angina pectoris)/myocardial injury (mainly termed as *myocardial enzyme elevation*), heart failure (HF)/left ventricular (LV) dysfunction, AF/atrial flutter (AFL), other arrhythmia/ECG abnormalities (ie, QTc prolongation), and cardiorespiratory arrest (CRA). We also assessed the temporal risk dynamics for the 90‐day mortality rate associated with the timing of SHS onset and its various manifestations. Subsequently, we conducted a comparative analysis of the impact on 90‐day mortality risk across different SHS manifestations, adjusting for a range of confounders. It is important to note that CRA was excluded from this analysis due to its direct association with an elevated mortality risk.

### Data Collection

Two authors (H.I. and B.H.) independently reviewed and identified terms describing adverse events associated with SHS in each patient. Events classified under nondefinitive categories, such as “possible” or “probable” diagnoses, were excluded from the analysis. The identified terms were cross‐checked for accuracy and consistency between the reviewers.

### Statistical Analysis

Variables with normal distributions were presented as mean±SD, and those with nonnormal distributions as medians with interquartile ranges. The Mann–Whitney *U* test and the χ^2^ test were used for comparing continuous and categorical variables, respectively. We calculated incidence rates with 95% CIs for the entire period and during 3 specific intervals (≤3 days, >3 to ≤10 days, and >10 to 30 days). The risk of death within 90 days was estimated using hazard ratios (HRs) with 95% CIs.

To illustrate how the hazard of death varied with the timing of the initial cardiac events, we plotted cubic spline curves for each SHS manifestation, adjusting HRs for age, sex, and baseline National Institutes of Health Stroke Scale score. We tested the overall association and nonlinearity between death and SHS, as well as each of its manifestations, using the Wald test. For the cubic spline curves, the selection of knot numbers was optimized within a range of 3 to 6, aiming for a balance between fewer knots and a lower Akaike information criterion. We also compared the adjusted HRs across the 3 intervals took the 0‐ to 3‐day period as the reference.

To assess the 90‐day mortality risks associated with the various SHS manifestations, we employed multivariate Cox proportional hazard models. The observation period began on the 31st day following stroke onset. The SHS manifestations of interest were ACS/myocardial injury, HF/LV dysfunction, AF/AFL, and other arrhythmia/ECG abnormalities. We adjusted for age, sex, initial National Institutes of Health Stroke Scale score, systolic blood pressure, hemoglobin levels, creatinine levels, and comorbidities (such as hypertension, diabetes, a history of stroke, AF, HF, and coronary artery disease). Missing values were imputed using the Multivariate Imputation by Chained Equations package in R (R Foundation for Statistical Computing, Vienna, Austria). A *P* value of <0.05 was considered statistically significant. The analyses were conducted using R software version 4.3.0, on a virtual network computer provided by VISTA.

## Results

### Patient Demographics

**Figure 1:**
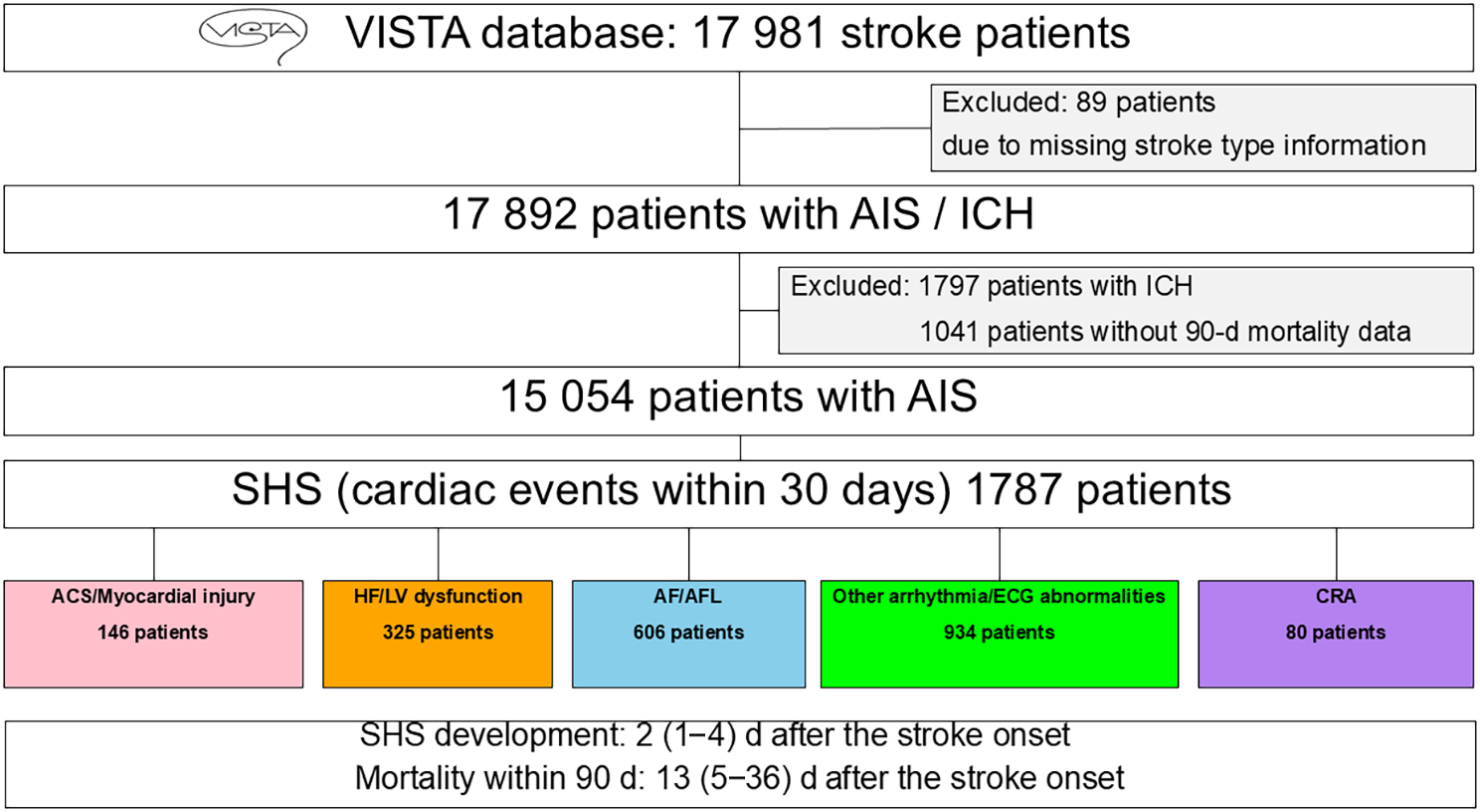
Study diagram. ACS indicates acute coronary syndrome; AF, atrial fibrillation; AFL, atrial flutter; AIS, acute ischemic stroke; CRA, cardiopulmonary arrest; HF, heart failure; ICH, intracranial hemorrhage; LV, left ventricular; SHS, stroke‐heart syndrome; and VISTA, Virtual International Stroke Trials Archive.

Figure 1 shows a flow diagram of the study. From a total of 17981individuals, 15 054 eligible patients were identified. Among 6548 terms related to adverse events, 248 were categorized as cardiac events. A total of 1787 patients, corresponding to 2091 adverse events, were recognized as cases of SHS. Patient demographics are outlined in Table 1. When compared with individuals without SHS, those diagnosed with SHS were older, predominantly women, exhibited more severe strokes, and had a higher prevalence of cardiovascular comorbidities.

**Table 1:** Patient Demographics

	Patients with SHS (n=1787)	Patients without SHS (n=13 267)	*P* value
Age, y, mean±SD [0]	73±11	69±13	<0.001
Male, n (%) [0]	937 (52)	7416 (56)	0.006
History of stroke, n (%) [0.3]	448 (25)	2857 (22)	<0.001
Baseline NIHSS score, mean±SD [25]	15±6	12±5	<0.001
Systolic blood pressure, mm Hg, mean±SD [0.6]	154±27	158±24	<0.001
Diastolic blood pressure, mm Hg, mean±SD [0.6]	81±17	85±15	<0.001
Creatinine, mg/dL, mean±SD [31]	1.0±0.36	0.95±0.33	<0.001
Hemoglobin, g/dL, mean±SD [38]	13±2	14±2	<0.001
Smoking [4]			<0.001
Nonsmoker, n (%)	750 (43)	5382 (42)	
Former smoker, n (%)	674 (39)	4083 (32)	
Current smoker, n (%)	307 (18)	3331 (26)	
Comorbidities			
Hypertension, n (%) [5]	1286 (72)	8415 (67)	<0.001
Diabetes, n (%) [0.05]	430 (24)	2807 (22)	0.006
History of AF, n (%) [14]	573 (33)	2591 (23)	<0.001
History of CAD, n (%) [33]	519 (38)	2200 (25)	<0.001
History of HF, n (%) [52]	259 (19)	496 (9)	<0.001

Numerical data are expressed as mean±SD or median (interquartile range; quartile 1–3). Categorical data are expressed as percentages and numbers. Numbers in brackets indicate missing rate. AF indicates atrial fibrillation; BP, blood pressure; CAD, coronary artery disease; HF, heart failure; NIHSS, National Institutes of Health Stroke Scale; and SHS, stroke‐heart syndrome.

### The Incidence and Mortality Rate of SHS

**Figure 2:**
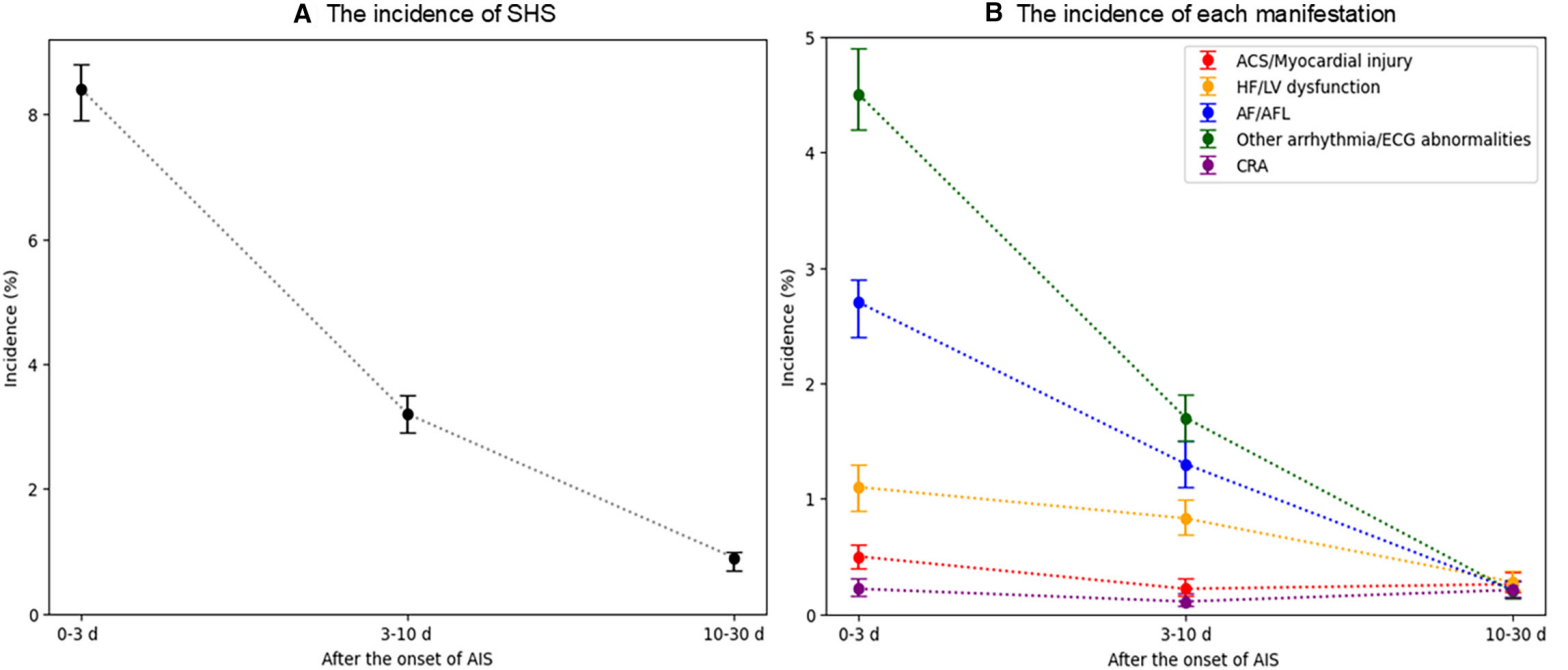
Incidence of SHS. **A**, Incidence of SHS. **B**, Incidence of each manifestation. Each plot is expressed as incidence (%) with 95% CI. ACS indicates acute coronary syndrome; AF, atrial fibrillation; AFL, atrial flutter; AIS, acute ischemic stroke; CRA, cardiopulmonary arrest; HF, heart failure; LV, left ventricular; and SHS, stroke‐heart syndrome.

Figure 2A illustrates the incidence trend of SHS. The overall incidence of SHS was 11.8% (95% CI, 11.3–12.3; Table 2). The median onset time for SHS was 2 (interquartile range, 1–4) days, with the majority of cases occurring within the first 10 days. Among patients with SHS, 620 patients (34%) died within the 90‐day period (Table 2).

**Table 2:** Development Day and Incidence of SHS

	Development day, Median (quartile 1–3)	Incidence, % (95% CI)	90‐day mortality rate, %
SHS	2 (1–4)	11.8 (11.3–12.3)	34
ACS/Myocardial injury	3 (2–11)	1.0 (0.86–1.2)	45
HF/LV dysfunction	4 (2–6)	2.2 (2.0–2.5)	56
AF/AFL	3 (2–4)	6.5 (6.1–6.9)	28
Other arrhythmia/ECG abnormalities	2 (1–4)	4.2 (3.9–4.6)	29
CRA	8 (2–18)	0.55 (0.44–0.69)	91

ACS indicates acute coronary syndrome; AF, atrial fibrillation, AFL, atrial flutter; CRA, cardiorespiratory arrest; HF, heart failure; LV, left ventricular; and SHS, stroke‐heart syndrome.

### The Incidence and Mortality Rate of Each Manifestation

Figure 2B shows the incidence trends of each SHS manifestation. The most prevalent manifestation was other arrhythmia/ECG abnormalities, with an incidence rate of 6.5% (95% CI, 6.1–6.9), followed by AF/AFL, HF/LV dysfunction, ACS/myocardial injury, and CRA (Table 2). While the majority of manifestations occurred within the first 10 days, the onset of CRA tended to occur later, at a median of 8 (interquartile range, 2–18) days after stroke. The mortality rate remained high across all manifestations, ranging from 28% for AF/AFL to 91% for CRA (Table 2).

Within the cohort identified with ACS/myocardial injury, a substantial majority, accounting for 91% (133/146 patients), were classified as having ACS. Similarly, among the patients with HF/LV dysfunction, 91% (297/325 patients), were categorized as having HF. Additionally, within the group diagnosed with AF/AFL, a significant proportion, 95% (574/606 patients), had a diagnosis with AF. Finally, of the patients with other arrhythmia/ECG abnormalities, 95% (883/934 patients) were categorized as having other arrhythmia.

### The Temporal Risk Dynamics for Death Based on SHS Onset Day

**Figure 3:**
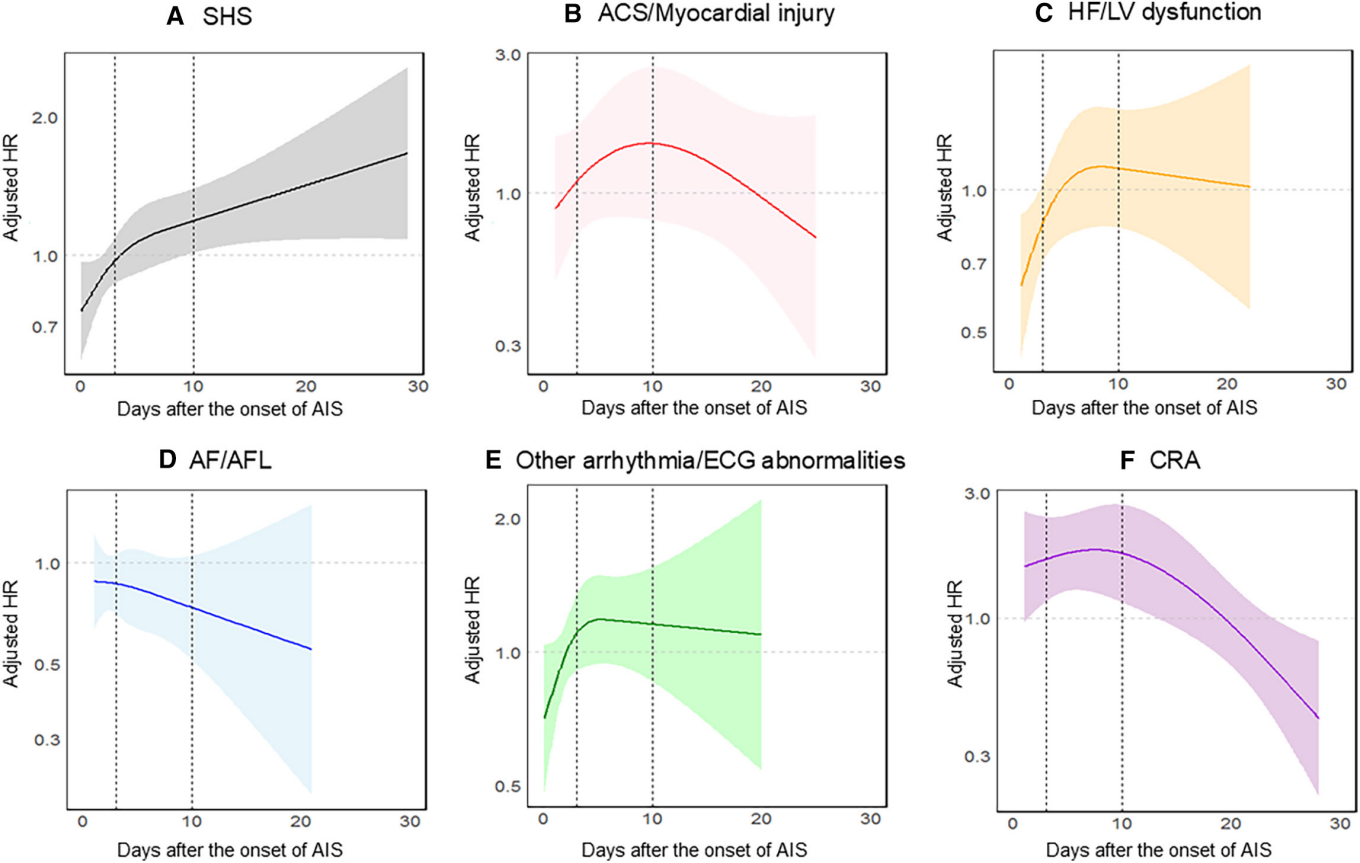
Temporal risk dynamics for dortality based on development day. **A**, SHS. **B**, ACS/myocardial injury. **C**, HF/LV dysfunction. **D**, AF/AFL. **E**, Other arrhythmia/ECG abnormalities. **F**, CRA. Each curve is expressed as adjusted hazard ratio with 95% CI. The dotted vertical lines on the left and right represent day 3 and day 10, respectively. ACS indicates acute coronary syndrome; AF, atrial fibrillation; AFL, atrial flutter; AIS, acute ischemic stroke; CRA, cardiopulmonary arrest; HF, heart failure; HR, hazard ratio; LV, left ventricular; and SHS, stroke‐heart syndrome.

Figure 3 showcased cubic spline curves that illustrate the 90‐day mortality risk associated with different timings of SHS onset. Based on the Akaike information criterion results, the optimal number of knots for all SHS manifestations was established at 3 (Table S1). The overall association and linearity between the mortality rate and SHS/each manifestation are shown in Table S2.

The mortality risk increased as the onset of SHS was delayed (Figure 3A). Specifically, patients who developed SHS between 10 and 30 days following stroke onset were found to have significantly higher mortality risks compared with those who developed SHS within the initial 0 to 3 days (Table 3).

**Table 3:** Comparison of 90‐Day Mortality Risks Across the Period of Development

	Adjusted HR (95% CI)	*P* value
SHS (N=1787)		
2df test		<0.001
≤3 d	1 (Reference)	
>3 to ≤10 d	1.08 (0.88–1.31)	0.461
>10 to 30 d*	1.84 (1.36–2.49)	<0.001
ACS/Myocardial injury (N=146)		
2df test		0.900
≤3 ds	1 (Reference)	
>3 to ≤10 d	1.05 (0.52–2.11)	0.875
>10 to 30 d	1.03 (0.49–2.17)	0.973
HF/LV dysfunction (N=325)		
2df test		0.300
≤3 d	1 (Reference)	
>3 to ≤10 d	1.42 (0.96–2.09)	0.082
>10 to 30 d	1.47 (0.79–2.73)	0.225
AF/AFL (N=606)		
2df test		0.300
≤3 d	1 (Reference)	
>3 to ≤10 d	0.78 (0.54–1.11)	0.210
>10 to 30 d	0.54 (0.19–1.48)	0.477
Other arrhythmia/ECG abnormalities (N=934)		
2df test		0.600
≤3 d	1 (Reference)	
>3 to ≤10 d	1.26 (0.94–1.66)	0.176
>10 to 30 d	1.43 (0.69–2.93)	0.233
CRA (N=80)		
2df test		0.007
≤3 d	1 (Reference)	
>3 to ≤10 d*	2.02 (1.01–4.04)	0.046
>10 to 30 d	0.77 (0.41–1.43)	0.411

HRs were adjusted by age, sex, and baseline NIHSS score. ACS indicates acute coronary syndrome; AF, atrial fibrillation, AFL, atrial flutter; CRA, cardiorespiratory arrest; HF, heart failure; HR, hazard ratio; LV, left ventricular; NIHSS, National Institutes of Health Stroke Scale; and SHS, stroke‐heart syndrome.

* Indicates statistical significance (*P*<0.05).

As for the temporal risk dynamics for mortality rate in each manifestation, the mortality risk in ACS/myocardial injury tended to peak at around 10 days after stroke (Figure 3B). For HF/LV dysfunction and other arrhythmia/ECG abnormalities, the mortality risk tended to peak between 5 and 10 days after stroke onset (Figure 3C and 3E). The risk of death for HF/LV dysfunction was found to be higher in the >3 to ≤10 days following the stroke onset, compared with the initial 0‐ to 3‐day period (*P*=0.082, as shown in Table 3).

Conversely, the mortality risk associated with AF/AFL demonstrated a decreasing trend over time (Figure 3D). This pattern suggests that the highest risk of death coincided with the onset of stroke, indicating an immediate impact on mortality rate following the stroke.

Similar to the pattern observed with ACS/myocardial injury, the mortality risk associated with CRA also reached its peak ≈10 days after the stroke onset (Figure 3F, Table 3). Beyond this peak period, the risk exhibited a tendency to decline, suggesting a specific window of elevated risk for patients experiencing CRA following stroke.

### Mortality Risks Across SHS Manifestations

**Figure 4:**
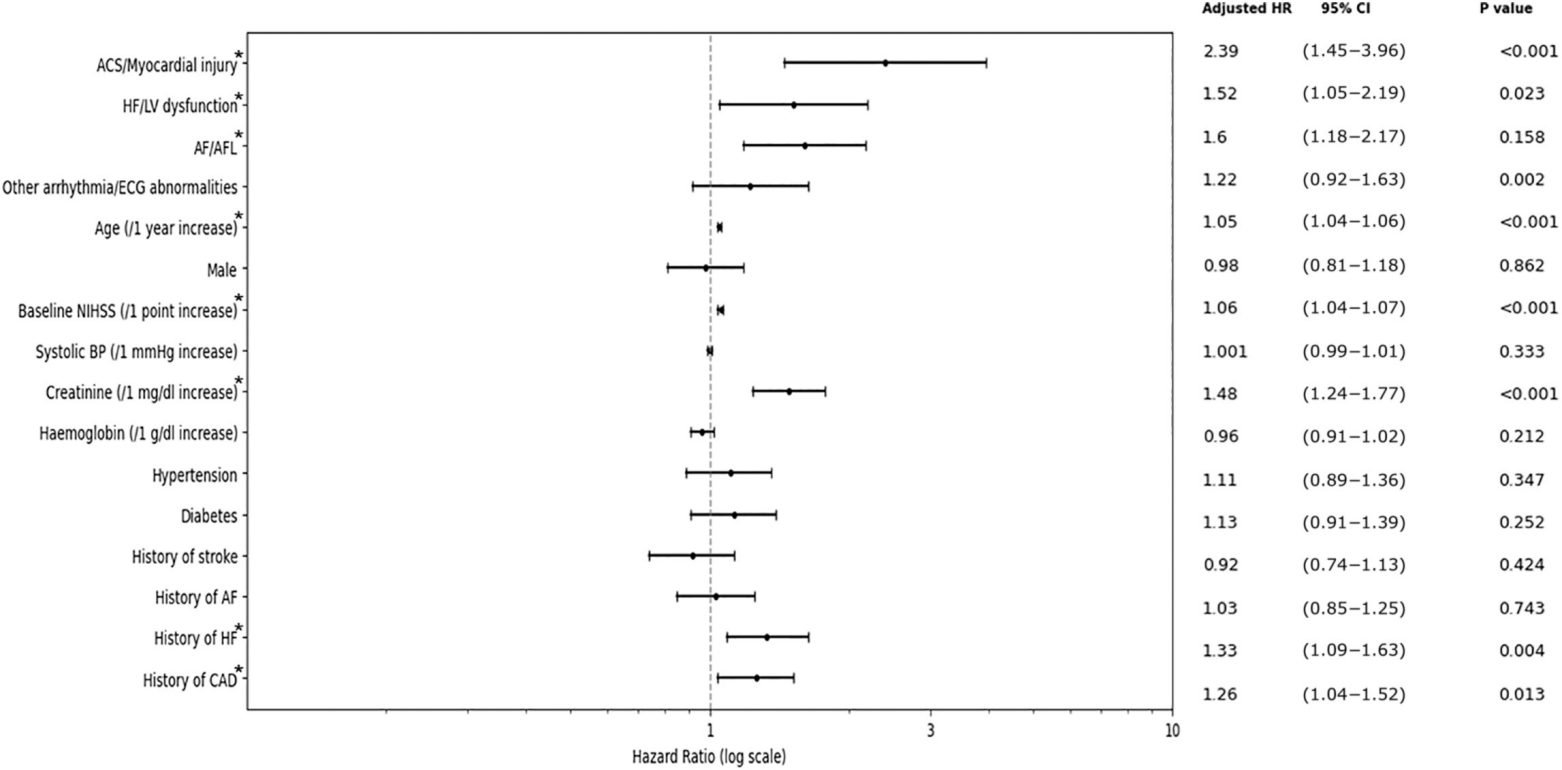
Mortality risks across SHS manifestations. Each plot is expressed as adjusted hazard ratio with 95% CI. * Statistical significance (*P*<0.05). ACS indicates acute coronary syndrome; AF, atrial fibrillation; AFL, atrial flutter; AIS, acute ischemic stroke; BP, blood pressure; CAD, coronary artery disease; CRA, cardiopulmonary arrest; HF, heart failure; HR, hazard ratio; LV, left ventricular; NIHSS, National Institutes of Health Stroke Scale; and SHS, stroke‐heart syndrome.

The results of 90‐day mortality risk across SHS manifestations following multivariate adjustment are shown in Figure 4. Among the manifestations of SHS, the development of ACS/myocardial injury, HF/LV dysfunction, and AF/AFL within 30 days following stroke were significantly associated with death (adjusted HR, 2.39 [95% CI, 1.45–3.96]; 1.52 [95% CI, 1.05–2.19]; and 1.60 [95% CI, 1.18–2.17], respectively). Additionally, other covariates such as age, baseline National Institutes of Health Stroke Scale score, creatinine level, and a history of HF and coronary artery disease were also significantly associated with an increased risk of death.

## Discussion

The key findings of this study are as follows: First, while the occurrence of SHS predominantly peaked within the first 3 days following stroke onset, the mortality risk tied to SHS demonstrated a gradual increase with delayed onset. Specifically, individuals developing SHS between 10 and 30 days after stroke had substantially higher mortality risk compared with those with SHS onset within the first 3 days after stroke. Second, certain SHS manifestations, notably HF/LV dysfunction, ACS/myocardial injury, and CRA, showed a trend of increasing mortality risk with delayed onset. In contrast, AF/AFL showed an immediate surge in mortality risk at the AIS onset, which lessened for cases emerging at later stages. Third, the development of ACS/myocardial injury, HF/LV dysfunction, and AF/AFL were significantly associated higher risk of death, compared with other SHS manifestations, excluding CRA, after multivariate adjustment.

### Temporal Risk Dynamics of Death Associated With the Timing of SHS Onset

The peak of incidence of SHS aligns with the surge in stress markers, such as catecholamines, suggesting that the acute stress responses triggered by AIS play a crucial role in the onset of SHS.^4^ Following this initial peak, the incidence for new cardiac events diminishes until day 14 following stroke, after which it shows a steady decline.^3^ Our findings reflect these observations and additionally found that mortality risk increases with delayed onset of SHS.

Several hypotheses may explain the observed outcomes. First, given that SHS involved a broad spectrum of manifestations varying in severity, it is plausible that milder manifestations occur more frequently immediately following the onset, whereas more severe conditions may manifest later. This concept is supported by our analysis, which showed a delayed peak in mortality risk for more severe manifestations such as HF/LV dysfunction and CRA. In contrast, we also infer that patients who developed SHS early on were likely predisposed to transient pathogeneses, such as arrhythmias and ECG abnormalities. These conditions are thought to be induced by self‐limiting neurogenic stressors, which are known to resolve over time.^15^, ^16^ Our hypothesis is further supported by observations indicating that the onset of other arrhythmia/ECG abnormalities preceded other clinical manifestations of SHS, with a median development time of 2 days after stroke onset.

Second, another potential hypothesis for the observed findings is the diagnostic delay in identifying SHS during the acute phase of stroke. The diagnosis of cardiac complications can be particularly challenging in this period due to the presence of neurological deficit or coma, obscuring the clinical presentations of cardiac issues.^17^ Moreover, previous studies have demonstrated that certain manifestations of SHS, such as Takotsubo syndrome and acute myocardial infarction, are asymptomatic in more than half of the affected patients.^10^, ^18^, ^19^ The development of SHS may be considered an outcome of a “stress test,” revealing previously undiagnosed cardiac conditions in patients with high‐risk cardiovascular comorbidities.^6^, ^20^, ^21^ Although our data did not specifically address the diagnostic process, it is reasonable to speculate that cases with higher risk may not have the positive results of “stress test” immediately recognized, potentially leading to a delay in the initiation of appropriate treatment.

### Temporal Risk Dynamics of Death in Each Manifestation

Our data demonstrated the variations in the temporal risk dynamics for death across different manifestations of SHS. Contrary to the mortality risk trends associated with ACS/myocardial injury, HF/LV dysfunction, and other arrhythmia/ECG abnormalities, AF/AFL, and CRA manifestations exhibited distinct temporal risk dynamics, diverging from the general SHS trend.

In the cases of AF/AFL, a trend of consistent decline in mortality risk over time was observed. This trend suggests that patients diagnosed with AF early on are more prone to exacerbations of preexisting AF or to instances in which AF was first detected at the onset of stroke. Previous studies suggested that the timing of AF detection reflected its subtype.^22^, ^23^ Preexisting AF (“known AF”) and AF first detected during the initial stroke presentation (“ECG‐detected AF”) were associated with a higher AF burden, more severe cardiovascular risk profiles, and poorer outcomes, in contrast with AF diagnosed later through extended ECG monitoring (AF detected after stroke).^24^, ^25^ The mortality risk trend observed in our study likely reflects these distinctions, highlighting the impact of early AF detection on patient outcomes.

CRA presented a unique temporal profile distinctly different from other SHS manifestations. In particular, the median onset of CRA was observed to be 8 days after stroke, followed by a downward trend in mortality risk after reaching its peak. Previous experimental studies have suggested a relationship between damage to the central autonomic network, such as the insular cortex, and sudden cardiac death, yet comprehensive clinical data are scarce.^26^, ^27^ A study conducted >3 decades ago, examining causes of death among patients with supratentorial infarction, found that sudden deaths typically occurred later, between 2 and 4 weeks after stroke onset, as opposed to deaths due to neurological reasons.^27^ Our findings incorporate into this historical observation and additionally revealed that the mortality risk associated with CRA tends to decrease for events occurring >10 days after the stroke onset. While the precise mechanisms underlying this trend remain to be fully understood, it may reflect the stabilization of the patient's overall health following the acute phase. Further research is warranted to explore whether this trend is a consistent finding across different cohorts.

### Clinical Implications

Our study highlights the variations of peak incidence across the different manifestations of SHS and the highest mortality risk associated with the timing of SHS development. The progressive increase in mortality risk associated with delays in the onset of SHS underlines the importance of early detection and intervention. Additionally, we have shown the substantial impact on mortality rate posed by ACS/myocardial injury, HF/LV dysfunction, and AF/AFL among the SHS manifestations. These findings highlight the critical need for clinicians to be vigilant in identifying and managing these specific complications. Recognizing the elevated mortality risk associated with these manifestations is of clinical importance.

### Limitations

Our study has several limitations. First, the identification of cardiac events within our analysis was based on the documentation of adverse events across various clinical trials included in the VISTA database, rather than direct examination of medical records. This method may lead to underestimation of the true incidence of SHS. This limitation highlights the need for using a more contemporary trial cohort for validation. Second, the retrospective design of our analysis imposed constraints on the availability of certain variables. Specifically, information regarding the anatomic locations of the stroke lesion, which have been shown to influence the incidence of SHS, could not be ascertained.^28^ Third, the data for our study were predominantly gathered from old clinical trials data, before the widespread adoption of high‐sensitivity cardiac troponin assays. Given that research using this assay has identified acute myocardial injury in approximately one fifth of patients,^29^, ^30^ our data set might not fully capture the incidence of this manifestation due to the era in which the data were collected.

## Conclusions

SHS is associated with a high risk of death, with a greater risk observed with delayed SHS onset. Among the SHS manifestations, ACS/myocardial injury, HF/LV dysfunction, and AF/AFL were associated with the highest risk of death post‐stroke.

## Sources of Funding

This study was funded by the Dowager Countess Eleanor Peel Trust, United Kingdom.

## Disclosures

Dr Dawson has received speaker fees from AstraZeneca, Bayer, Boehringer Ingelheim, Bristol‐Myers Squibb, Daiichi Sankyo, Medtronic, and Pfizer, as well as a travel fee from MicroTransponder Inc. He also received an investigator‐initiated research funding grant from the Stroke Association UK. Dr Lip reports consultant and speaker for BMS/Pfizer, Boehringer Ingelheim, Daiichi‐Sankyo, and Anthos. No fees are received personally. He is a National Institute for Health and Care Research senior investigator and co‐principal investigator of the Atrial Fibrillation Integrated Approach in Frail, Multimorbid, and Pollymedicated Older People project on multimorbidity in AF (grant agreement No. 899871), TARGET project on digital twins for personalized management of atrial fibrillation and stroke (grant agreement No. 101136244) and Apixaban for Reduction in Stroke and Other Thromboembolic Events in Atrial Fibrillation (ARISTOTLE) project on artificial intelligence for management of chronic long term conditions (grant agreement No 101080189), which are all funded by the European Union's Horizon Europe Research and Innovation program. The remaining authors have no disclosures to report.

## Supporting information

Tables S1‐S2

## VISTA‐Acute Steering Committee members

K. R. Lees (Chair), A. Alexandrov, P. M. Bath, E. Bluhmki, N. Bornstein, C. Chen, L. Claesson, J. Curram, S. M. Davis, H‐C. Diener, G. Donnan, M. Fisher, M. Ginsberg, B. Gregson, J. Grotta, W. Hacke, M. G. Hennerici, M. Hommel, M. Kaste, P. Lyden, J. Marler, K. Muir, C. Roffe, R. Sacco, A. Shuaib, P. Teal, N. Venketasubramanian, N. G. Wahlgren, and S. Warach.
