# Supplementary material for: Mortality Risk in Patients With Cardiac Complications Following Ischemic Stroke: A Report From the Virtual International Stroke Trials Archive
Source: J Am Heart Assoc. 2024 Nov 22;13(23):e036799. doi: 10.1161/JAHA.124.036799 (PMC11681577; doi:10.1161/JAHA.124.036799)
Supplement: Supplementary file 1 — Tables S1‐S2 [file JAH3-13-e036799-s001.pdf]

SUPPLEMENTAL MATERIAL

Table S1. Comparison of Akaike Information Criterion Values for Different Number of Knots in Spline Curves

	Knot			
	3	4	5	6
SHS	6,498	6,499	6,499	6,498
ACS/Myocardial injury	422	420	422	422
HF/ LV dysfunction	1,285	1,283	1,286	1,288
AF/AFL	1,528	1,528	1,527	1,527
Other arrhythmia/ ECG abnormalities	2,715	2,717	2,716	2,718
CRA	524	524	526	528

ACS, acute coronary syndrome; AF, atrial fibrillation, AFL, atrial flutter; CRA, cardiorespiratory arrest; ECG, electrocardiogram; HF, heart failure; LV, left ventricle; SHS, stroke-heart syndrome

Table S2. The overall association and non-linearity between mortality and SHS/each manifestation

	P-values	
	The overall association	Non-linearity
SHS	0.005	0.262
ACS/Myocardial injury	0.366	0.161
HF/ LV dysfunction	0.144	0.069
AF/AFL	0.605	0.934
Other arrhythmia/ ECG abnormalities	0.178	0.104
CRA	0.002	0.103

ACS, acute coronary syndrome; AF, atrial fibrillation, AFL, atrial flutter; CRA, cardiorespiratory arrest; ECG, electrocardiogram; HF, heart failure; LV, left ventricle; SHS, stroke-heart syndrome
